# Correlation between Kidney Uptake at [18F]FDG PET/CT and Renal Function

**DOI:** 10.3390/jpm14010040

**Published:** 2023-12-28

**Authors:** Francesco Dondi, Antonio Rosario Pisani, Nicola Maria Lucarelli, Maria Gazzilli, Anna Talin, Domenico Albano, Dino Rubini, Nicola Maggialetti, Giuseppe Rubini, Francesco Bertagna

**Affiliations:** 1 Nuclear Medicine, Università Degli Studi di Brescia and ASST Spedali Civili Brescia, 25123 Brescia, Italy; f.dondi@outlook.com (F.D.); anna.talin@gmail.com (A.T.); domenico.albano@unibs.it (D.A.); francesco.bertagna@unibs.it (F.B.); 2 Section of Nuclear Medicine, Interdisciplinary Department of Medicine, University of Bari “Aldo Moro”, Piazza Giulio Cesare 11, 70124 Bari, Italy; antoniorosario.pisani@uniba.it (A.R.P.); nicola.maggialetti@uniba.it (N.M.); giuseppe.rubini@uniba.it (G.R.); 3 Nuclear Medicine, ASL Bari—P.O. Di Venere, 70012 Bari, Italy; 4 Department of Precision Medicine, University of Campania “L. Vanvitelli”, 80138 Naples, Italy; dinoru95@hotmail.it

**Keywords:** CKD, chronic kidney disease, PET/CT, positron emission tomography, [^18^F]FDG

## Abstract

Different insights into the connection between kidney [^18^F]fluorodesoxyglucose ([^18^F]FDG) uptake at positron emission tomography/computed tomography (PET/CT) and renal function have been proposed in the past. The aim of this study was therefore to assess the presence of a correlation between these two parameters. Kidney uptakes were assessed and compared to the creatinine (Cr) values and estimated glomerular filtration rate (EGFR) among different classes of renal functional impairment or kidney status. A total of 339 patients and 385 different PET/CT scans were included in this study. Significant correlations between kidney uptakes and renal function parameters were reported in most of the groups studied, with the exception of patients with Cr < 1.2 mg/dL and subjects with a kidney transplantation. Strong concordance in the assessment of renal parenchymal uptakes between the different readers was reported. To conclude, strong correlations for renal [^18^F]FDG uptake with Cr levels and the EGFR were reported, with the exception of the group of patients with a Cr value < 1.2 mg/dL and for the group with a kidney transplantation.

## 1. Introduction

Chronic kidney disease (CKD) is a clinical syndrome caused by an irreversible change in renal function usually characterized by a slowly progressive evolution [1]. This condition has a higher prevalence in the adult population and is associated with different other pathological entities, such as an increased risk of cardiovascular disease and death [1]. Many different causes can contribute to the development of CKD; however, the most frequent are hypertension and diabetes, chronic glomerulonephritis or pyelonephritis, polycystic kidney disease, Alport’s disease, autoimmune diseases, congenital malformations and the use of anti-inflammatory drugs [1,2].

CKD is diagnosed when an estimated glomerular filtration rate (EGFR) lower than 60 mL/min/1.73 m^2^ has been present for at least three months or in the presence of an alteration in the renal structure. The disease can be classified into five different stages according to the impairment of the EGFR, which represents the rate at which the glomerulus filters plasma to produce urine and it is used to diagnose, stage and manage CKD [3]. These parameters cannot be measured directly so it is estimated based on the blood concentration of an endogenous filtration marker that is usually represented by creatinine (Cr), the most commonly used for this purpose [3]. Cr originates from muscle mass and diet through meat proteins, is freely filtered by the glomerulus and also secreted by the renal tubules [3]. The Kidney Disease: Improving Global Outcomes (KDIGO) CKD guidelines recommend the use of the EGFR for the determination of the disease because of its simplicity, availability, cost-effectiveness and accuracy in predicting renal function in standard conditions. The estimating equation recommended by the KDIGO work group for the assessment of the EGFR in adults is the CKD Epidemiology Collaboration (CKD-EPI) [3,4].

[^18^F]fluorodesoxyglucose ([^18^F]FDG) positron emission tomography/computed tomography (PET/CT) is a hybrid imaging modality that in the last few years has been gaining more and more attention, due to its ability to evaluate a high amount of different conditions, both neoplastic or benign [5,6,7]. [^18^F]FDG is a glucose analog able to identify tissues with high glycolytic activity and it is excreted by the kidneys with incomplete reabsorption in the proximal renal tubules [8]. Nevertheless, the usefulness of [^18^F]FDG PET/CT for the assessment and the evaluation of different renal conditions has been reported in the past. Interestingly, it has been underlined that renal tissue is able to concentrate [^18^F]FDG and that this uptake is related to the degree of sensitivity to insulin [9]. Moreover, a theoric model has been proposed that showed that the more severe the renal failure is, the more overestimated the standardized uptake value (SUV) is, unless the renal failure indirectly impairs tissue sensitivity to insulin and hence glucose metabolism [10]. Furthermore, some insights on the role of PET/CT imaging for the evaluation and the follow-up of renal allograft rejection and early evaluation of treatment response have been proposed in the literature [11,12,13,14]. PET/magnetic resonance (PET/MR) performed with [^18^F]FDG also revealed a possible role to estimate renal function in terms of the EGFR and effective renal plasma flow (ERPF) [15]. Moreover, given the fact that, as mentioned, a higher cardiovascular risk is present in patients with CKD and that CKD is associated with myocardial metabolic changes, it has been reported that [^18^F]FDG PET/CT could have the ability to investigate preclinical myocardial abnormalities in these patients [16]. Similarly, PET/CT was proposed as a valuable diagnostic tool for verifying and quantifying accelerated atherosclerosis secondary to CKD in patients on hemodialysis, with changes in tracer uptake that appeared to be accelerated in these subjects [17]. Interestingly, it has also been reported that [^18^F]FDG PET/CT could assess the presence of inflammation in the carotid arteries, revealing therefore that renal transplantation may confer an anti-inflammatory effect on carotid atherosclerosis [18]. In addition, hybrid molecular imaging with [^18^F]FDG is known to have high sensitivity for the evaluation of a wide range of inflammatory diseases and in this setting, it demonstrated its role for the diagnosis of the cause of fever of unknown origin (FUO) in CKD subjects on hemodialysis [19,20,21]. Similarly, PET/CT has also demonstrated its role for the evaluation of patients with autosomal dominant polycystic kidney disease and suspected cyst infection, with high diagnostic performances [22].

Some studies on the ability of CKD to modify the uptake of [^18^F]FDG during a PET/CT scan have also been published with heterogeneous results. In some cases, it has been reported that the impairment of renal function does not significantly compromise the clearance of the background activity of [^18^F]FDG in PET imaging [23]. In contrast, other papers instead observed that patients with CKD have a higher physiological tracer uptake in the liver and blood pool [24]. However, it has been demonstrated that CKD patients on hemodialysis show a significantly higher physiological [^18^F]FDG uptake in the soft tissues, spleen and the blood pool compared to normal subjects [25]. Despite that, it was, however, suggested that CKD and subsequent renal failure do not require an adjustment in PET/CT protocols and that the standard protocol times should also be used in patients with renal failure [26].

The main purpose of our study was therefore to search for a possible correlation between renal uptake at [^18^F]FDG PET/CT and renal functional parameters in both normal subjects and patients with CKD.

## 2. Materials and Methods

### 2.1. Patients Selection

In order to select patients suitable for inclusion in the present study, we retrospectively analyzed our databases to find subjects with CKD submitted to our center to undergo [^18^F]FDG PET/CT for any reason. The interval time of this study was between January 2011 and May 2023. The exclusion criteria were as follows: (1) age under 18 years, (2) presence of pathological conditions affecting kidneys (e.g., cancers, inflammation, polycystic kidney disease) that made it impossible to assess kidney [^18^F]FDG uptake, (3) absence of serum Cr values in the 7 days before or after the PET/CT scan and (4) presence of liver disease, affecting the value of the SUVmax. After applying such criteria, 224 patients with CKD were included. Moreover, 115 subjects without CKD who underwent [^18^F]FDG PET/CT in our institution for various conditions were arbitrarily selected as the controls, with the same exclusion criteria mentioned before applied.

Ethical review and approval were waived for this study due to its retrospective design, according to local laws and to the ethics committees of our center. Informed consent was obtained from all individual participants included in this study. Information about gender and age was collected for all the subjects.

For all the patients included in this study, data about serum Cr levels and estimated glomerular filtration rate (EGFR) calculated with the CKD-EPI formula [3] were collected. Based on the EGFR, patients were also classified between the 5 stages of CKD. Moreover, in the case of patients undergoing different PET/CT scans, the evolution of these values at the time of the scan was also assessed and, in particular, dCr was calculated as the difference between the value of Cr at the second evaluation and the value at the first evaluation. Similarly, dEGFR was calculated as the difference between the EGFR at the time of the second PET/CT scan and the value at the first scan.

### 2.2. [^18^F]FDG PET/CT Acquisition and Interpretation Protocol

A blood glucose level lower than 150 mg/dL was required for all the patients before undergoing [^18^F]FDG PET/CT and they fasted for at least 6 hours before tracer injection. Patients voided before imaging acquisition, no oral or intravenous contrast agents were administrated or bowel preparation used for any patient. Blood glucose levels and the use of insulin replacement therapy at the moment of the scan were collected. An activity of 3.5–4.5 MBq/Kg of [^18^F]FDG was intravenously administered and images were acquired at least 60 ± 10 min after injection from the skull basis to the mid-thigh on a Discovery ST or Discovery 690 PET/CT tomograph (General Electric Company, GE, Milwaukee, Wisconsin) with standard parameters (CT: 80 mA, 120 kV; PET: 2.5–4 min per bed position, PET step of 15 cm). Reconstructions were performed with a 256 × 256 matrix and a 60 cm field of view. On the Discovery 690 tomograph, time of flight (TOF) and point spread function (PSF) algorithm were used for the reconstruction of images, with filter cut-off 5 mm, 18 subsets and 3 iterations. For the Discovery ST tomograph, an ordered subset expectation maximization (OSEM) algorithm with filter cut-off 5 mm, 21 subsets and 2 iterations was applied. 

**Figure 1:**
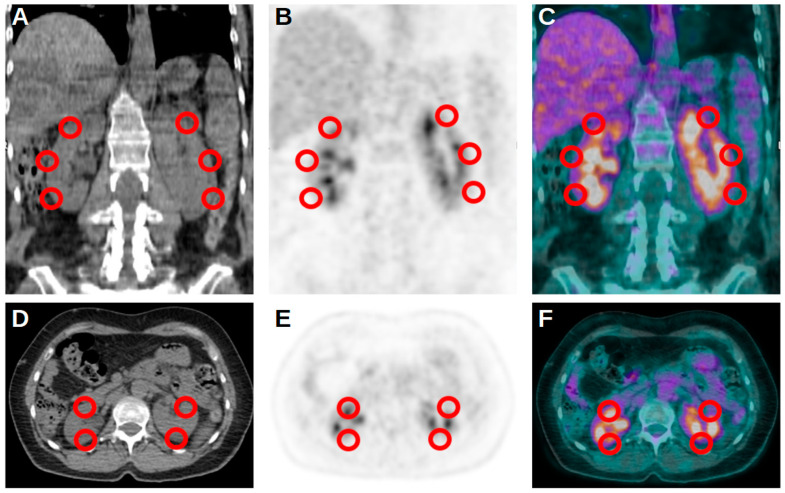
Representative and schematic figures in coronal (**A**–**C**) and axial (**D**–**F**) projection of the 5 VOIs used for the assessment of kidney uptake at [^18^F]FDG PET/CT.

PET/CT images were visually and semiquantitatively analyzed by two experienced nuclear medicine physicians. First of all, renal uptake for each kidney was calculated as the average of the SUVmax of 5 spheric volumes of interest (VOI) for each kidney, as presented in Figure 1. To obtain the total kidney uptake of a single patient (K), a mean of all its renal average uptakes was calculated. Moreover, the SUVmax of the liver was calculated using a spheric VOI with 1 cm diameter placed at the VIII hepatic segment from transaxial PET images. A similar VOI was used to obtain the SUVmax of the blood pool at the aortic arch from transaxial PET images, paying attention to not involve the vessel’s walls. These two values were used to calculate a ratio with K (KL and KBP, respectively). Again, in the case of subjects with multiple PET/CT scans, dK, dKL and dKBP were calculated as the difference between the value at the second imaging evaluation and the value at the first scan.

### 2.3. Statistical Analysis

Statistical analyses were performed using SPSS Software version 29.0.1.0 for Macintosh (New York, NY, USA). The descriptive analysis of categorical variables was carried out comprising the calculation of simple and relative frequencies. Numeric variables were described as mean, SD, minimum and maximum values (range). 

Firstly, intraclass correlation coefficient (ICC) was used to calculate concordance and to evaluate the reproducibility of the assessment of renal uptakes at PET/CT between the two readers. To assess the correlation between Cr levels and PET/CT parameters, Pearson’s test was applied. The same analysis was also performed to search for a correlation between eGFR levels and PET/CT parameters. Similarly, the search for the presence of a possible correlation between dCr and dEGFR with PET/CT uptakes, the same statistic test was applied. The aforementioned analyses were performed considering all the cohort of the study, only patients without a kidney transplantation, the group of transplanted patients and also considering cut-off values of 1.2 mg/dL for Cr (the upper limit of normal Cr value in our institution) and of 60 mL/min/1.73 m^2^ for the EGFR. For all the aforementioned statistics, a *p*-value < 0.05 was considered significant.

Renal PET/CT semiquantitative parameters were also compared between patients, dividing them on the basis of the aforementioned values of 1.2 mg/dL for Cr and 60 mL/min/1.73 m^2^ for the EGFR. These analyses were performed using T-test and were applied to the total cohort of patients and to the group of patients without kidney transplantation. No analyses were taken into account for transplanted subjects, since most of them had Cr > 1.2 mg/dL and/or EGFR > 60 mL/min/1.73 m^2^. Moreover, ANOVA test was used to search for significant differences in terms of renal uptakes at PET/CT between the different classes of renal impairment based on the EGFR. T-test and Chi-square test were used to compare blood glucose levels and the use of insulin replacement therapy between the different classes of patients included in the study. Again, a *p*-value < 0.05 was considered significant for all these statistics.

## 3. Results

A total of 339 patients were included in our study (190 male, 56%). The mean age was 65, standard deviation (SD) 14 and range 18–92 years. Some patients underwent more than a single [^18^F]FDG PET/CT study and therefore the total number of scans included in this study were 385. Most of the subjects had 2 functioning kidneys (303, 89.4%), 17 (5.0%) patients were functional monokidney, while 19 (5.6%) had a kidney transplantation. In particular, 17 subjects (5.0%) had renal tracer uptake only on the transplanted kidney, while 2 (0.6%) had uptake on both native and transplanted kidneys (Table 1).

The mean Cr value of our cohort was 2.1 (SD 2.35, range 0.36–11.1) and at the moment of the PET/CT scans, 231 patients (60.0%) had a Cr < 1.2 mg/dL, while the remaining 154 (40.0%) subjects had a Cr ≥ 1.2 mg/dL. In terms of the EGFR, the mean value of all the cohort was 63.8 (SD 38.5, range 4.2–139.7), with 226 (58.7%) subjects with a value ≥ 60 mL/min/1.73 m^2^ at the time of PET/CT and the remaining 159 (41.3%) patients with an EGFR < 60 mL/min/1.73 m^2^.

After classifying the patients based on CKD stage, 133 (39.2%) of them had a stage I disease, 85 (25.1%) had a stage II disease, 33 (9.7%) had a stage III disease, 27 (8.0%) had a stage IV disease and, lastly, 61 (18.0%) subjects had a stage V disease.

Blood glucose levels were collected at the moment of the PET/CT scan; the mean value was 113 mg/dL, range was 73–147 and SD was 21. No significant differences were reported for this parameter between patients with CKD and the controls (*p*-value 0.079), between subjects with a Cr value below and above 1.2 mg/dL (*p*-value 0.077) and between subjects with an EGFR below and above 60 mL/min/1.73 m^2^ (*p*-value 0.098). Furthermore, at the moment of the PET/CT scan, 133 patients were using insulin replacement therapy; no significant differences in terms of the use of this therapy were underlined between patients with CKD and the controls (*p*-value 0.773), between subjects with a Cr value below and above 1.2 mg/dL (*p*-value 0.913) and between subjects with an EGFR below and above 60 mL/min/1.73 m^2^ (*p*-value 0.676).

**Figure 2:**
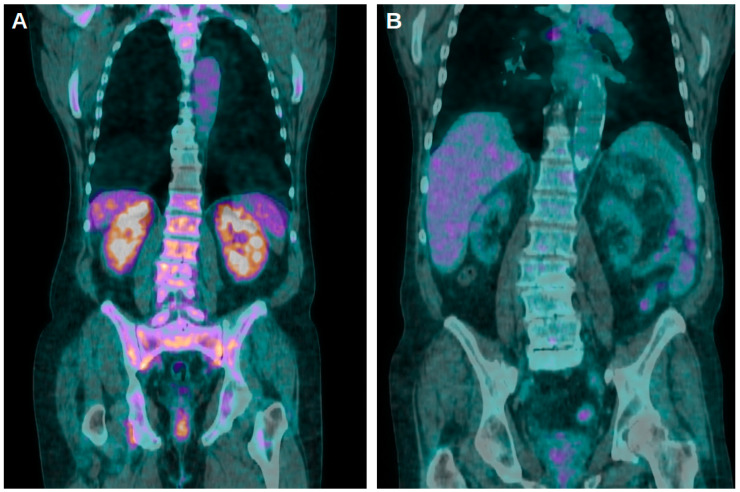
(**A**) Coronal fused [^18^F]FDG PET/CT images of a patient with a Cr value of 1.41 mg/dL and an EGFR of 56.9 mL/min/1.73 m^2^ at the moment of the scan. Stage of CKD was 3 and K, KL and KBP values were 8.85, 1.97 and 3.69, respectively. (**B**) Four years later, the same subjects again underwent a PET/CT scan: Cr raised to 9.12 mg/dL, EGFR dropped to 5.80 mL/min/1.73 m^2^ and the stage of CKD was 5. K, KL and KBP were 1.82, 0.79 and 1.01, respectively.

A strong concordance in the assessment of renal uptakes between the two readers was revealed by ICC analysis (r 0.848, *p*-value < 0.001). In the total cohort of the patients included in this study, we reported significant negative correlations for Cr values with K, KL and KBP. Significant positive correlations were instead underlined for the EGFR with K, KL and KBP. Significant negative correlations were also demonstrated for dCr and dEGFR with dK, dKL and dKBP. When focusing only on the group of subjects without kidney transplantation, the same insights were confirmed and, again, Cr, dCr and dEGFR revealed a significant negative correlation with the PET/CT semiquantitative parameters, while the EGFR confirmed its positive correlation with K, KL and KBP. Moreover, when considering only transplanted patients, no significant correlations were underlined between the renal function parameters and PET/CT results. Similarly, no significant correlations between these data were reported in the group of subjects with a Cr < 1.2 mg/dL at the time of PET/CT. In contrast, when performing these analyses for subjects with a Cr ≥ 1.2 mg/dL at the time of PET/CT, negative significant correlations for Cr with K, KL and KBP, for dCr with dK, dKL and dKBP and for dEGFR with dK, dKL and dKBP were confirmed. Significant positive correlations were confirmed for the EGFR and PET/CT semiquantitative parameters. These findings on the presence of significant negative correlations for Cr, dCR and dEGFR and on the presence of a positive correlation for the EGFR with the PET/CT semiquantitative parameters were also confirmed when dividing the patients on the basis of an EGFR value of 60 mL/min/1.73 m^2^ (Table 2; Figure 2).

Statistically significant differences in K, KL and KBP values were reported in all the cohort of our study, when dividing the patients based on a Cr value of 1.2 mg/dL. These findings were confirmed when dividing them based on an EGFR value of 60 mL/min/1.73 m^2^. Again, the same analysis performed in the cohort of transplanted patients confirmed the aforementioned findings. No analyses were performed in the group of subjects with a transplanted kidney, since it was a limited sample and most of them had Cr ≥ 1.2 mg/dL and/or EGFR > 60 mL/min/1.73 m^2^. Lastly, statistically significant differences in terms of K, KL and KBP were underlined between the different classes of EGFR impairment (Table 3).

## 4. Discussion

It is known that kidney function is one of the main determinants of [^18^F]FDG excretion and, as mentioned before, different studies have tried to investigate a possible influence of renal failure on the interpretation of PET/CT imaging and on renal uptake with heterogeneous results [8,23,27].

Our findings revealed statistically significant K, KL and KBP values between different classes of renal impairment, between groups with different Cr values and between different EGFR values for the total cohort of patients and in the case of subjects without kidney transplantation. These analyses could not be performed for transplanted patients since most of them had a significant impairment of renal function. Moreover, statistically significant negative correlations between Cr values and renal [^18^F]FDG uptake in the total cohort of patients included in this study and in most of the subgroups evaluated during the analyses were reported. Similarly, in most of the cases, statistically significant positive correlations between the EGFR and renal uptakes were reported. A possible explanation for these findings is the fact that an impairment of renal function, related to an increase in Cr values, could determine a reduction in glucose metabolic activity in the kidneys and therefore a reduction in tracer uptake. Interestingly, these insights were also confirmed when analyzing the temporal evolution of Cr blood levels and the evolution of renal uptakes in the case of patients with multiple PET/CT scans. In contrast, a significant negative correlation was reported between dEGFR and the evolution of renal PET uptakes; however, in order to interpret this different finding compared to the aforementioned positive correlation of the EGFR, it is important to consider that only a small sample of patients were studied with more than a single PET/CT scan.

No significant correlations were reported in the group of patients that had a kidney transplantation and in the group of subjects with a Cr value < 1.2 mg/dL. The first finding is in concordance with the findings reported by Jadoul et al. [28], who revealed that the uptake of [^18^F]FDG by renal allografts is not significantly impacted by CKD. The fact that no significant correlations were underlined for patients with a blood Cr value < 1.2 mg/dL is an insight that could be taken into account in the day life interpretation of [^18^F]FDG PET/CT scans, since the possible presence of reduced renal uptake could reflect the presence of an unknown impairment of the renal function of the patient. It is worth underlining that we reported a significant correlation between kidney uptakes and the EGFR in the group of patients that had a value ≥ 60 mL/min/1.73 m^2^; however, this insight was not confirmed in the group of subjects that had a Cr value < 1.2 mg/dL. By definition, these two subgroups should contain most of the same patients as a low Cr and high EGFR are correlated and since the formula used to calculate the EGFR includes Cr. A possible explanation for this interesting finding could be the fact that according to the CKD-EPI formula, the calculation of the EGFR should include other parameters, such as sex and age, resulting therefore in some differences between Cr and EGFR values. In fact, Cr only expresses a serum marker and is not used to classify the degree of renal functional impairment; instead, this partition is based on the EGFR, which represents the estimated function of the kidney and is therefore a more reliable tool. 

As previously mentioned, kidneys are organs that can excrete [^18^F]FDG through the urinary tract; therefore, the assessment of renal tracer uptake could be impaired by the presence of radioactive urine in the renal pelvis [27,29]. Our findings, however, revealed a strong concordance in the assessment of renal parenchymal uptakes between the two readers, a finding that is in concordance with what was previously underlined by Jadoul et al. [30] when assessing the repeatability and reproducibility of the quantification of [^18^F]FDG uptake by kidney allograft. As mentioned, it has also been reported that kidney [^18^F]FDG uptake is related to the degree of sensitivity to insulin and that renal failure indirectly impairs this sensitivity and therefore glucose metabolism [9,10]. We considered this phenomenon in our study and no significant differences in terms of blood glucose levels at the moment of the PET/CT scan or the use of insulin replacement therapy were reported between the different groups of subjects, therefore strengthening our findings.

Our work is not without limitations; first of all, its retrospective design. Furthermore, the problem of the quantification of renal uptake, which could be influenced by the presence of radioactive urine, has been described in the discussion, even if our data underlined a good concordance between the two readers. Moreover, some analyses have been performed in a restricted group of patients; therefore, wider samples should be used to confirm our findings.

## 5. Conclusions

In conclusion, a strong correlation for renal [^18^F]FDG uptake with the Cr levels and EGFR was reported, with exceptions for the group of patients with a Cr value < 1.2 mg/dL and for the group with a kidney transplantation. High agreement between the two readers in the assessment of renal tracer uptakes was underlined.

## Figures and Tables

**Table 1:** Characteristics of the 339 patients included in the study.

Characteristics	Number (%)
Age (mean ± SD, range)	65 ± 14, 18–92
Gender	
Male	190 (56.0%)
Female	149 (44.0%)
Kidneys Status	
Functional monokidney	17 (5.0%)
Two functioning kidneys	303 (89.4%)
Transplanted	19 (5.6%)
Only transplanted kidney uptake	17 (5.0%)
Uptake on transplanted and native kidneys	2 (0.6%)
Cr (mg/dL) (mean ± SD, range)	2.1 ± 2.35, 0.36–11.1
Cr at the time of PET/CT	
<1.2 mg/dL	231 (60.0%)
≥1.2 mg/dL	154 (40.0%)
EGFR (mL/min/1.73m2) (mean ± SD, range)	63.8 ± 38.5, 4.2–139.7
EGFR at the time of PET/CT	
≥60 mL/min/1.73 m^2^	226 (58.7%)
<60 mL/min/1.73 m^2^	159 (41.3%)
CKD stage	
1	133 (39.2%)
2	85 (25.1%)
3	33 (9.7%)
4	27 (8.0%)
5	61 (18.0%)

SD: standard deviation; Cr: creatinine; EGFR: estimated glomerular filtration rate; PET/CT: positron emission tomography/computed tomography; CKD: chronic kidney disease.

**Table 2:** Correlation between renal functional parameters and PET/CT parameters.

	*p*-Value	Rho
All patients (*n* = 339)		
Cr-K	<0.001	−0.33
Cr-KL	<0.001	−0.34
Cr-KBP	<0.001	−0.38
EGFR-K	<0.001	0.32
EGFR-KL	<0.001	0.35
EGFR-KBP	<0.001	0.42
dCr-dK	<0.001	−0.50
dCr-dKL	0.005	−0.41
dCr-dKBP	0.005	−0.42
dEGFR-dK	<0.001	−0.54
dEGFR-dKL	<0.001	−0.61
dEGFR-dKBP	<0.001	−0.59
Non-transplanted patients (*n* = 320)		
Cr-K	<0.001	−0.33
Cr-KL	<0.001	−0.34
Cr-KBP	<0.001	−0.39
EGFR-K	<0.001	0.34
EGFR-KL	<0.001	0.38
EGFR-KBP	<0.001	0.45
dCr-dK	<0.001	−0.53
dCr-dKL	0.005	−0.46
dCr-dKBP	<0.001	−0.54
dEGFR-dK	<0.001	−0.56
dEGFR-dKL	<0.001	−0.65
dEGFR-dKBP	<0.001	−0.72
Transplanted patients (*n* = 19)		
Cr-K	0.095	−0.32
Cr-KL	0.111	−0.31
Cr-KBP	0.114	−0.31
EGFR-K	0.867	−0.03
EGFR-KL	0.863	0.03
EGFR-KBP	0.562	0.12
dCr-dK	0.528	−0.26
dCr-dKL	0.926	0.04
dCr-dKBP	0.944	−0.03
dEGFR-dK	0.211	−0.49
dEGFR-dKL	0.391	−0.35
dEGFR-dKBP	0.541	−0.25
Cr < 1.2 mg/dL (*n* = 231)		
Cr-K	0.199	−0.80
Cr-KL	0.119	−0.10
Cr-KBP	0.110	−0.10
EGFR-K	0.635	0.03
EGFR-KL	0.234	0.06
EGFR-KBP	0.128	0.10
dCr-dK	0.793	0.16
dCr-dKL	0.663	−0.26
dCr-dKBP	0.879	0.09
dEGFR-dK	0.638	0.28
dEGFR-dKL	0.981	−0.01
dEGFR-dKBP	0.731	−0.21
Cr ≥ 1.2 mg/dL (*n* = 154)		
Cr-K	<0.001	−0.46
Cr-KL	<0.001	−0.49
Cr-KBP	<0.001	−0.47
EGFR-K	<0.001	0.56
EGFR-KL	<0.001	0.54
EGFR-KBP	<0.001	0.56
dCr-dK	<0.001	−0.55
dCr-dKL	0.007	−0.43
dCr-dKBP	0.006	−0.44
dEGFR-dK	<0.001	−0.59
dEGFR-dKL	<0.001	−0.64
dEGFR-dKBP	<0.001	−0.60
EGFR ≥ 60(mL/min/1.73 m^2^) (*n* = 226)		
Cr-K	<0.001	−0.33
Cr-KL	<0.001	−0.34
Cr-KBP	<0.001	−0.38
EGFR-K	<0.001	0.32
EGFR-KL	<0.001	0.46
EGFR-KBP	<0.001	0.42
dCr-dK	<0.001	−0.50
dCr-dKL	0.005	−0.41
dCr-dKBP	0.005	−0.42
dEGFR-dK	<0.001	−0.54
dEGFR-dKL	<0.001	−0.61
dEGFR-dKBP	<0.001	−0.59
EGFR < 60(mL/min/1.73 m^2^)(*n* = 159)		
Cr-K	<0.001	−0.44
Cr-KL	<0.001	−0.49
Cr-KBP	<0.001	−0.46
EGFR-K	<0.001	0.51
EGFR-KL	<0.001	0.53
EGFR-KBP	<0.001	0.56
dCr-dK	<0.001	−0.55
dCr-dKL	0.007	−0.42
dCr-dKBP	0.006	−0.44
dEGFR-dK	<0.001	−0.59
dEGFR-dKL	<0.001	−0.64
dEGFR-dKBP	<0.001	−0.60

Cr: creatinine; K: kidney uptake; KL: kidney to liver ratio; KBP: kidney to blood pool ratio; EGFR: estimated glomerular filtration rate; dCr: differential Cr value; dK: differential kidney uptake; dKL: differential kidney to liver ratio; dKBP: differential kidney to blood pool ratio; dEGFR: differential estimated glomerular filtration rate; *n* = number.

**Table 3:** Correlation between class of renal impairment, creatinine and EGFR with PET/CT parameters.

	K	*p*-Value	KL	*p*-Value	KBP	*p*-Value
All patients (*n* = 339)						
Cr		<0.001		<0.001		<0.001
<1.2 mg/dL (*n* = 231)	4.63		1.78		2.36	
≥1.2 mg/dL (*n* = 154)	3.35		1.13		1.44	
EGFR		<0.001		<0.001		<0.001
≥60 (mL/min/1.73 m^2^) (*n* = 226)	4.66		1.79		2.38	
<60 (mL/min/1.73 m^2^) (*n* = 159)	3.33		1.14		1.44	
Non-transplanted patients (*n* = 320)						
Cr		<0.001		<0.001		<0.001
<1.2 mg/dL ( *n* = 231)	4.66		1.79		2.37	
≥1.2 mg/dL (*n* = 154)	3.16		1.03		1.32	
EGFR		<0.001		<0.001		<0.001
≥60 (mL/min/1.73 m^2^) ( *n* = 226)	4.69		1.79		2.39	
<60 (mL/min/1.73 m^2^) (*n* = 159)	3.15		1.04		1.32	
EGFR ( *n* = 339)		<0.001		<0.001		<0.001
≥90 (mL/min/1.73 m^2^) ( *n* = 133)	4.76		1.85		2.49	
60–89 (mL/min/1.73 m^2^) ( *n* = 85)	4.51		1.68		2.21	
30–59 (mL/min/1.73 m^2^) ( *n* = 33)	4.36		1.46		1.90	
15–29 (mL/min/1.73 m^2^) ( *n* = 27)	3.14		1.16		1.41	
<15 (mL/min/1.73 m^2^) ( *n* = 61))	2.69		0.91		1.14	

Cr: creatinine; K: kidney uptake; KL: kidney to liver ratio; KBP: kidney to blood pool ratio; EGFR: estimated glomerular filtration rate; *n* = number.

## Data Availability

Data available on request due to privacy/ethical restrictions.
